# Model-Based Damage Localization Using the Particle Swarm Optimization Algorithm and Dynamic Time Wrapping for Pattern Recreation

**DOI:** 10.3390/s23020591

**Published:** 2023-01-04

**Authors:** Ilias Zacharakis, Dimitrios Giagopoulos

**Affiliations:** 1 Department of Mechanical Engineering, University of Western Macedonia, 50100 Kozani, Greece; 2 Department of Mechanical Engineering, Aristotle University of Thessaloniki, 54124 Thessaloniki, Greece

**Keywords:** damage detection, damage localization, vibration-based, metaheuristic algorithms, dynamic time wrapping

## Abstract

Vibration-based damage detection methods are a subcategory of Structural Health Monitoring (SHM) methods that rely on the fact that structural damage will affect the dynamic characteristic of a structure. The presented methodology uses Finite Element Models coupled with a metaheuristic optimization algorithm in order to locate the damage in a structure. The search domains of the optimization algorithm are the variables that control a parametric area, which is inserted into the FE model. During the optimization procedure, this area changes location, stiffness, and mass to simulate the effect of the physical damage. The final output is a damaged FE model which can approximate the dynamic response of the damaged structure and indicate the damaged area. For the current implementation of this Damage Detection Framework, the Particle Swarm Optimization algorithm is used. As an effective metric of the comparison between the FE model and the experimental structure, Transmittance Functions (TF) are used that require output only acceleration signals. As with most model-based methods, a common concern is the modeling error and how this can be surpassed. For this reason, the Dynamic Time Wrapping (DTW) algorithm is applied. When damage occurs in a structure it creates some differences between the Transmittance Functions (TF) of the healthy and the damaged state. With the use of DTW, the damaged pattern is recreated around the TF of the FE model, while creating the same differences and, thus, minimizing the modeling error. The effectiveness of the proposed methodology is tested on a small truss structure that consists of Carbon-Fiber Reinforced Polymer (CFRP) filament wound beams and aluminum connectors, where four cases are examined with the damage to be located on the composite material.

## 1. Introduction

Damage detection and localization in structural systems is a high-priority task during inspection in many branches of civil and mechanical engineering. In this field, but also in the general field of Structural Health Monitoring (SHM), the research community is embracing technological advancements. One example is the application of Machine Learning (ML) for SHM systems [1,2,3,4], which rely on experimentally measured data or artificially collected data to properly train the ML model for classification. Other methods have also presented promising results, such as the eigen perturbation methodologies [5,6,7].

Recently, a Damage Detection Framework was presented [8] that is using vibration measurements in conjunction with Finite Element Models. The current work is advancing the same framework by addressing the issue of modeling errors between the physical structure and the FE model, which can be a serious concern in complex structures. The Damage Detection Framework is based on the model updating technique using optimization algorithms that fall into the category of computational intelligence and take advantage of the available computational power of modern computers. Several works can be found in the literature on this subject [9,10,11,12,13], using a large number of different optimization algorithms such as Genetic Algorithms [14], Covariance Matrix Adaptation Evolution Strategy (CMA-ES) evolutionary algorithms [15], and Artificial Bee Colony (ABC) [16], but also other algorithms such as the Particle Swarm Optimization (PSO) [17] and the Bat algorithm [18]. A common practice among these past works is the use of simplified FE models, using beam elements, with the methods applied on truss-like structures. On the other hand, few of the past researchers have incorporated more detailed FE models also using plate elements [19,20,21]. Additionally, two review articles [22,23] can give a broader view to the reader on the specific subject. A common characteristic among these works is the number of parameters of the optimization procedure where the stiffness and/or mass of every element (or group of elements) is set as a parameter to be updated. This leads to updating the global stiffness and/or mass matrix of the structure, where the difference between the updated and the original matrix reveals the affected damaged areas. If the same procedure was applied to detailed FE models, it could lead to an increase in the computational cost. This would be the result of the increase of the optimization parameters (updating the properties of each element or group of elements) and, in parallel, the computational cost of the FE model would be higher than the simplified FE model.

Whether a model-based methodology is using simplified or detailed FE models, the accuracy and applicability of those methods are highly correlated with the percentage of modeling error with the corresponding structure. The major novelty of the current procedure is the minimization of the effect of the modeling error by recreating the pattern of the damaged experimental measurements of the structure based on the dynamic response of the FE model. While this methodology is embedded and tested inside the current Damage Detection Framework, it would be possible for other model-based methodologies to embed and benefit from it as well.

The presented methodology can be applied to complex structures consisting of multiple parts and different materials. It has the advantage of a fixed number of optimization parameters; thus, only the computational cost of the FE model itself affects the performance between different structures. However, this computational cost must always be taken into consideration as detailed FE models in exceptionally large structures can have an impact, which can be addressed by applying appropriate model reduction methods. The framework embeds a metaheuristic algorithm, whereby for the current implementation the Particle Swarm Optimization (PSO) algorithm [24] was selected. The search space of the optimization algorithm consists of six parameters in total that control a parametric damaged area which is inserted into the FE model. Four parameters control the exact location, along with two parameters that control the material properties of the specific damaged area. During the optimization, this area changes location, stiffness, and mass to simulate the effect of the real damage in a physical structure. As a result, only a submatrix of the stiffness and mass matrices is changing. A similar approach that locally alters the material properties has been also demonstrated [25] on a Carbon Fiber Reinforced Polymer (CFRP) composite plate using Genetic Algorithms (GA). As will be presented in the following sections, this implementation can be applied in detailed FE models that might contain beam, shell, and solid elements as well.

The metric of comparison between the structure and the FE model plays a pivotal role in the accuracy and applicability of the methods in use. In the current work, the Transmittance Function (TF) [26] is used, which is a sensitive metric regarding structural damages [27]. The TF has the advantage of requiring only output experimental data.

The modeling error between the physical structure and the FE model can sometimes be a serious concern in model-based methods. In order to minimize this error and develop an optimal FE model for the structure, the Covariance Matrix Adaptation Evolution Strategy (CMA-ES) algorithm [28,29,30] was selected, that has been applied successfully at linear and non-linear FE updating problems [31,32] in the past.

Even after the model updating procedure, there is a possibility that the modeling error still exists in a higher percentage than it is acceptable for the current application. This is most probable when the examined structure is complex and might include multiple parts, materials, and joints. To surpass the effect of the modeling error as much as possible, the Dynamic Time Wrapping (DTW) algorithm is applied. The DTW is a popular algorithm that was first introduced for speech recognition [33], and it has found many applications such as for clustering and classification of electrocardiograms (ECG) [34], online signature recognition [35], and process monitoring [36]. Although with a different implementation, the DTW has been also applied in fault diagnostics and monitoring [37,38,39]. In the current work, the DTW is applied in order to recreate the pattern of the experimental damaged structure around the FE model. The recreated pattern is a Transmittance Function (TF) curve. The difference between the TF curves obtained from DTW and the FE model is the same as the one obtained from the healthy and damaged structure in an experiment. As such, the new curve is used on the framework as a damaged TF, which minimizes the error of the FE model. Other researchers in the past have also noted that these types of SHM systems do not need to locate and/or quantify the damage with extreme accuracy [40]. Thus, the inserted damaged area on the FE model needs to simply contain the damage of the physical structure and indicate the affected area.

The proposed methodology relies upon and advances the previously presented Damage Detection Framework [8] and, as such, has the following advantages. First, it is a model-based damage detection method using vibration measurements with output-only information. Second, it can be applied to detailed FE models of any shape, and structures with multiple parts and different materials. Third, with a properly configured optimization algorithm, only a fixed number of six optimization parameters are needed to locate the damage using a detailed FE model. Finally, fourth, with the use of the DTW, the effect of the modeling error, which could otherwise cause inaccurate results or even prevent the applicability of the methodology, is now minimized by recreating the damaged pattern.

This work is presented as follows. Section 2 describes the implementation of the metaheuristic optimization algorithms for this SHM method. Section 3 includes the background of the proposed methodology, and the use of Transmittance Functions, which is an efficient output-only metric. Furthermore, it includes the use of the Dynamic Time Wrapping algorithm to recreate the damage pattern and disregard the initial modeling error. In order to validate the proposed methodology, Section 4 presents the application on an experimental truss structure consisting of CFRP beams where four damage cases were created. The results show the robustness of the proposed Damage Detection Framework and the capabilities of locating damage in the structure. Finally, the conclusions are summarized in Section 5.

The following work is an advancement of the Damage Detection Framework [8], which was presented in the past by the authors. Therefore, the reader is encouraged to also refer to this previous work.

## 2. Metaheuristic Algorithms

As a model-based detection method, the first step is to develop an optimal Finite Element Model with an accurate dynamic response for the examined structure. Many algorithms can tackle this model updating procedure in order to develop an accurate FE model, and in the current work the Covariance Matrix Adaptation Evolution Strategy (CMA-ES) algorithm is used.

The proposed Damage Detection Framework, on the other hand, was embedded with the Particle Swarm Optimization (PSO) algorithm, which has been found more suitable for this task. Other optimization algorithms could also be used for this task, although the selection should be made with caution. For example, both CMA-ES and PSO can perform in the presented damage–detection framework in a simple single-part model. However, in a more complex model consisting of multiple parts and multiple materials, the CMA-ES could not perform as intended due to its internal selection and sampling mechanism.

### 2.1. Covariance Matrix Adaptation Evolution Strategy (CMA-ES)

The CMA-ES [28,29,30] is a general-purpose, population-based, stochastic, derivative-free algorithm. A brief explanation of CMA-ES’s main steps can be found in Algorithm 1.
**Algorithm 1.** Main steps of CMA-ES algorithm.Initialize distribution parameters**While** termination criterion is not met **do**  Sample population from the multivariate normal distribution  Evaluate the objective function for each parameter set  Update the multivariate normal distribution based on a percentage (50% in this case) of the best parameter sets.**End**The optimal solution is found for the parameter set that corresponds to the minimum objective function

The FE Model can be described by the set of parameters $\underline{\theta }\in R$, where $g\left(\underline{\theta }\right)$ is the model prediction for the set of parameters $\underline{\theta }$, while y corresponds to the dynamic experimental measurements. The objective functions, which will be minimized, can be formulated as the sum of the normalized sum of square errors J:

(1) $$J\left(\underline{\theta }\right)=\frac{1}{n}\sum_{i=1}^{n}\frac{\sum_{j=1}^{m}{\left(g_{ij}\left(\underline{\theta }\right)-y_{ij}\right)}^{2}}{\sum_{j=1}^{m}{\left(y_{ij}\right)}^{2}}$$

The Transmittance Function will act as a metric of comparison between the physical structure and the FE model.

As such, $g(\underline{\theta }),\;y$ are the FE models and experimental Transmittance Functions, with the subscript *j* to denote the frequency step and *i* denoting the index of the Transmittance curve, while *n* is the total number of Transmittance Functions and *m* is the total number of frequency steps.

### 2.2. Particle Swarm Optimization (PSO) Algorithm

One of the main parts of the Damage Detection Framework is the Particle Swarm Optimization (PSO) algorithm. This algorithm is used in order to find the best set of variables of the damaged area in the FE model. The PSO is a population-based algorithm that belongs to the subarea of Swarm Intelligence in the Computational Intelligence category, and was introduced by Kennedy and Eberhart [24,41].

The variant of the PSO that is being used in the current manuscript is the initial version [24], while adding the Inertia Weight [42] into the equation that controls the velocities of each particle. Additionally, two subversions can be found in the literature; the GBEST and LBEST [41]. Each particle has a specific velocity, where a portion of its velocity is attributed to the best solutions of the other particles in the swarm. In the GBEST version, the velocity is influenced by the best solution found by all other particles, while in the LBEST version each particle’s velocity is influenced by the best solution from a number of its nearest particles (called its neighborhood). The LBEST version was chosen in this work.

The initial swarm (population), $Pop=\{p_{1},p_{2},\ldots ,p_{n}\}$, is sampled randomly and consists of n number of particles,$p_{i}\in {\mathbb{R}}^{k}$ for i=1,2,..,n, where k is the number of parameters to be optimized. Each particle $p_{i}$ has a position, $x_{i}^{t}$, and velocity vector, $v_{i}^{t}$, at the given time step t.

The velocities and positions of each particle are updated in every cycle based on the following rules:

(2) $$v_{i}^{t+1}=\underset{\mathrm{Inertia}}{\underset{︸}{w\cdot v_{i}^{t}}}+\underset{\mathrm{Cognitive}}{\underset{︸}{c_{1}\cdot R1_{\left(i,\;i\right)}\cdot (pbest_{i}^{t}-x_{i}^{t})}}+\underset{\mathrm{Social}}{\underset{︸}{c_{2}\cdot R2_{\left(i,\;i\right)}\cdot (lbest_{i}^{t}-x_{i}^{t})}}$$

(3) $$x_{i}^{t+1}=x_{i}^{t}+v_{i}^{t+1}$$

where w is the inertia weight and $c_{1},c_{2}$ the acceleration coefficient, which are all defined prior to the start of the algorithm. R1, R2 are two k×k diagonal matrices with diagonal elements sampled at each iteration from a uniform random distribution with values from 0 to 1. Furthermore, pbest is a vector containing the best parameter values of the corresponding particle and lbest is a vector containing the best parameter values from the neighborhood particles. The neighborhood N is a fraction of the total number of particles n.

Considering Equation (2), the Inertia term carries the particle into its previous direction, the Cognitive Part is the force that pulls the particle towards its personal best position, and the Social Part is the force that drags the particle towards the best position known from its neighborhood particles [43].

The objective function, G, is discussed in the following Section 3. Algorithm 2 summarizes the main steps of the Particle Swarm Optimization algorithm.
**Algorithm 2.** Main steps of the PSO Algorithm.Set *w, c_1_, c_2_, n* and variable boundsRandomly generate the initial swarm while enforcing the variable bounds**While** termination criterion is not met **do****for** each particle *i* **do**  Evaluate the objective functions  **if** $G(p_{i}^{t})<G(pbest_{i})$ **then** $pbest_{i}\leftarrow p_{i}^{t}$  $lbest_{i}^{t}=\min(pbest_{\mathrm{neighbors}}^{t})$  Update velocity, **Equation (2)**  Update position, **Equation (3)**, while enforcing the variable bounds**End for****End while**The optimal solution is found as the parameter set that corresponds to the minimum objective function

## 3. Damage Detection Framework

The main parts of the Damage Detection Framework are similar to the framework which was presented by the authors in the past [8]. A short description of these parts is included in Section 3.1, Section 3.2 and Section 3.3 while the reader is encouraged to also refer to the previous work.

The modeling error can play a pivotal role in the model-based damage detection methods. As the modeling error rises, most of the model-based methods become less accurate and, after one point, even not applicable, including [8]. In Section 3.4 a new method is proposed, as an extension of the previous method [8], to minimize the effect of the modeling error for the purpose of damage detection, making the method applicable even in cases where the model cannot be corrected further.

### 3.1. Description of the Damage Detection Framework

The relationship between a healthy structure, S, and the corresponding Optimal FE Model, M, could be written in a general form as:

(4) $$M=S+e_{1}$$

When a damage occurs at the structure, Equation (4) can be transformed as:

(5) $$M_{dam}=S_{dam}+e_{2}\;\Rightarrow \;M+dM=S+dS+e_{2}$$

where $S_{dam}$ and $M_{dam}$ represent the damaged structure and a corresponding damaged FE Model. Additionally, parameters $e_{1},\;e_{2}$ are the error between the structure and the FE Model. The error two parameters are not equal as the damaged FE Model may not be able to reach the same accuracy as the healthy FE Model. This is due to the possible creation of nonlinear behavior of the damaged area in the physical structure where the newly created damaged FE Model is only linear.

The overall goal of the Damage Detection Framework is to find the dM, which is highly correlated with dS. The term dS represents the change of the structure, or else the damage. The term dM is the change that the Optimal FE Model, M, must make in order to approximate the $S_{dam}$. In the current work, dM includes the location of the damaged area. While an approximation of the material mechanical parameters for this damaged area is included in the dM as well, they must not be interpreted as a quantification of the damage. This approximation is limited to providing an insight into the type of damage, e.g., loss of stiffness in case of a crack. Exact quantification of the damaged properties is not possible, mainly due to the modeling errors.

In other words, the framework searches for local changes in the material properties in different locations of the FE Model while trying to approximate the dynamic experimental measurements of the damaged structure.

The Optimal Finite Element Model (M) of the examined structure can be described by the equation of motion [44]:

(6) $$M\ddot{x}+C\dot{x}+Kx=F$$

where F is the external excitation and $\ddot{x},\;\dot{x},\;x$ represent the acceleration, velocity, and displacement vectors. M, C, K are the mass, damping, and stiffness matrix, accordingly, and so the model can be fully described by M(M,C,K). Assuming that damage in a structure will affect the mass and stiffness matrices, the major target is to find the appropriate d M and d K that results in the corresponding dM, and, therefore, the $M_{dam}$ that approximates the damaged structure $S_{dam}$.

The difference in the mass and stiffness matrices is found by inserting a damaged area into the FE model and changing its material mechanical properties. The task of finding the location and properties of this area is assigned at the PSO algorithm. The search domain of the optimization algorithm includes six parameters in total. The first two represent the percentage change of the Elastic Modulus and Density,$\left\{p_{E},p_{D}\in \mathbb{R}:(0,UB]\right\}$ where *UB* is the selected upper bound. The final Elastic Modulus and Density of the damaged area are calculated using Equations (7) and (8):

(7) $${\vec{E}}_{dam}=p_{E}\cdot {\vec{E}}_{part}$$

(8) $$D_{dam}=p_{D}\cdot D_{part}$$

where the subscript *dam* indicates the material properties (modulus, density) of the damaged area and *part* is the material properties of the part in which the area is inserted. The Elastic Modulus is expressed as a vector in case the material is not a standard isotropic and might have moduli at other directions (such as a Carbon-Fiber Reinforced Composite material).

The exact location of the damaged area which is inserted into the FE model is controlled by the remaining four parameters. The vector L(P,X,Y,Z) describes the location of the inserted area, where P,$\left\{P\in \mathbb{R}:[0,1]\right\}$, represents the part of the FE Model in a multi-part structure and X,Y,Z,$\left\{X,Y,Z\in \mathbb{R}:[0,1]\right\}$, corresponds to the local coordinates expressed as a fraction of the total dimensions of the specific part chosen by P. As such, the search space of the PSO consists of these six parameters $\left(p_{E},p_{D},P,X,Y,Z\right)$. It is worth noting that the final material parameters of the damaged area do not quantify the real damage as their value is also affected by the size of the inserted area and, most importantly, by the modeling error. The inserted damaged area can be created in any 3D model, with either 2D shell elements or 3D solid elements.

The import, manipulation and export of the FE Model were implemented in MATLAB. For the evaluation of the dynamic response of the FE model, the commercial solver MSC NASTRAN was selected.

### 3.2. Transmittance Function

The Transmittance Function (TF) [26] is expressed as the ratio of the Cross-Spectral (CSD),$S_{rs}$, over the Auto-Spectral Density (PSD),$S_{rr}$, between two vibration response signals calculated from Equation (9).

(9) $$T_{rs}(\omega )=\frac{S_{rs}(\omega )}{S_{rr}(\omega )}=\frac{{\ddot{x}}_{r}(\omega ){\ddot{x}}_{s}^{*}(\omega )}{{\ddot{x}}_{r}(\omega ){\ddot{x}}_{r}^{*}(\omega )}$$

where $\ddot{x}(\omega )$ is the Fourier transform of the acceleration signal with *ω* as the frequency. Furthermore, ${\ddot{x}}^{*}(\omega )$ is the complex conjugate of $\ddot{x}(\omega )$ and subscripts *r, s* denote the degrees of freedom of the structure. The TF has been found to be a sensitive method for Structural Health Monitoring purposes [26,45], while requiring only output measurements.

If *w* is the number of all the acceleration sensors which are placed on the structure, assuming triaxial accelerometers, the complete TF matrices can be formulated by calculating all the possible TF combinations (axis-specific):

(10) $$T^{X}={\left[\begin{array}{cccc} 1 & T_{12}^{X} & \cdots & T_{1w}^{X}\\ T_{21}^{X} & 1 & & T_{2w}^{X}\\ \vdots & & \ddots & \vdots \\ T_{w1}^{X} & T_{w2}^{X} & \cdots & 1 \end{array}\right]}_{w\times w}T^{\Upsilon }={\left[\begin{array}{cccc} 1 & T_{12}^{\Upsilon } & \cdots & T_{1w}^{\Upsilon }\\ T_{21}^{\Upsilon } & 1 & & T_{2w}^{\Upsilon }\\ \vdots & & \ddots & \vdots \\ T_{w1}^{\Upsilon } & T_{w2}^{\Upsilon } & \cdots & 1 \end{array}\right]}_{w\times w}\;T^{Ζ}={\left[\begin{array}{cccc} 1 & T_{12}^{Ζ} & \cdots & T_{1w}^{Ζ}\\ T_{21}^{Ζ} & 1 & & T_{2w}^{Ζ}\\ \vdots & & \ddots & \vdots \\ T_{w1}^{Ζ} & T_{w2}^{Ζ} & \cdots & 1 \end{array}\right]}_{w\times w}$$

From the matrices of Equation (10) only the unique combinations are used from the Damage Detection Framework and, as such, only the upper triangular part of the T matrices is taken into consideration.

The TF is an output-only metric between two response signals, Equation (9), and, as such, there is no obligation to record the input excitation during testing in the structure. On the part of the FE model, while an excitation needs to be provided as input, an artificial excitation can also be used as it will not affect its TFs.

### 3.3. Objective Function

The objective function is formulated between the TFs of the experimental structure and the FE model, using the Pearson Correlation coefficient. It is a measure of the linear correlation between two data sets that take values between −1 and 1. Considering two sets of data A, B, with equal length, N, and μ, σ as the mean value and standard deviation of each set, the Pearson Correlation coefficient can be calculated with Equation (11).

(11) $$Pearson\;Correlation\;Coefficient:\;\rho (A,B)=\frac{1}{N-1}\sum_{i=1}^{N}\left(\frac{A_{i}-\mu_{A}}{\sigma_{A}}\right)\left(\frac{B_{i}-\mu_{B}}{\sigma_{B}}\right)$$

A value of ρ=1 indicates that there is a perfect linear correlation between the two data sets, ρ=−1 indicates a negative linear correlation, while ρ=0 indicates that there is a nonlinear relationship but without providing any further details.

In the current minimization problem, the best solution is considered the one with the best linear correlation between the damaged structure and the FE model. The final objective function, G, can be formed with Equation (12) as the mean value of the errors (from the linear correlation) of the Pearson coefficients between the experimental measurements of the damaged structure, $TF^{DEXP}$, and the FE model, $TF^{FE}$, where *A* denotes the total number of Transmittance Functions used with *i* = 1, 2,…,A.

(12) $$Objective\;Function,\;G=\frac{1}{A}\sum_{i=1}^{A}\left[1-\rho \left(T_{i}^{DEXP},T_{i}^{FE}\right)\right]$$

### 3.4. Dynamic Time Wrapping (DTW)

Dynamic Time Wrapping (DTW) is an algorithm to measure similarities between two data series (e.g., time-series) and it was initially developed for speech recognition [33]. Consider two time signals, C and Q, with similar features across their duration. Their features appear in the same sequence, but some of them can be in different moments such as after a small delay. The DTW wraps the features of each signal in a non-linear matter across the time domain iteratively and calculates the wrapping path and its distance. This procedure stops when the wrapping path with the minimum distance is found. The most commonly used distance metric for this algorithm is the sum of Euclidian distance between each data point of the signals.

First, create the distance matrix, Equation (13), between the two signals where each element is the distance between all elements, Equation (14). Iteratively, using the distance matrix, the DTW finds the paths for each signal that minimizes the sum of all distances; see Equation (15).

(13) $$.D=\left[\begin{array}{ccccc} d_{11} & & \cdots & & d_{1q}\\ & \ddots & & & \\ \vdots & & d_{ij} & & \vdots \\ & & & \ddots & \\ d_{q1} & & \cdots & & d_{qq} \end{array}\right].$$

(14) $$d_{ij}={\left(C_{i}-Q_{j}\right)}^{2}\;i,j=1,2,\ldots ,q$$

(15) $$DTW_{\mathrm{Distance}}=\sum d_{ij}\;i,j=1,2,\ldots ,q$$

In the present work, the DTW will be used within a different context. The algorithm will be used to calculate the wrapping path between a TF curve on the healthy state of the experimental structure and the FE model. Basically, the wrapping path is the index of each of the two arrays that is being altered in order to minimize Equation (15). The path of the healthy TF curve is the shift of each frequency point in order to wrap around the FE model’s TF curve. This shift is calculated on the frequency domain. As such, exactly the same path can be used on the damaged TF curve of the experimental structure. A damage in an experimental structure that might affect only a portion of a TF curve. Using this wrapping path, a new curve will be created which will be the reconstructed damaged TF curve. As the healthy TF curve will be wrapped around the TF of the model, the same differences that exist between the healthy and damaged TF will now be created between the recreated damaged TF and the TF of the FE model. The unaffected parts of the experimental TF will remain unaffected but shifted in the frequency domain, and the affected parts will follow the pattern that exists between the two states of the experimental structure. The procedure is summarized in Algorithm 3.
**Algorithm 3.** Main steps to reconstruct the damaged curve using the DTW.Calculate the $TF_{Healthy}$, $TF_{Damage}$ and $TF_{FEM}$Apply the DTW between FEM and Healthy$\left[DTW_{\mathrm{Distance}},PATH_{Healhty},PATH_{FEM}]\leftarrow \;DTW(TF_{Healthy},TF_{FEM}\right)$Reconstruct the new curves$TF_{RecHealthy}=TF_{Healthy}\left(PATH_{Healhty}\right)$$TF_{RecFEM}=TF_{FEM}\left(PATH_{FEM}\right)$$TF_{RecDamage}=TF_{Damage}\left(PATH_{Healhty}\right)$

**Figure 1:**
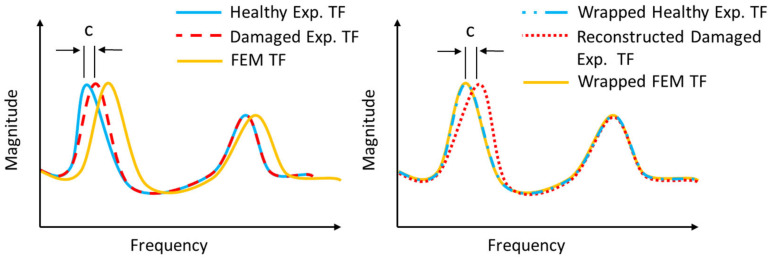
Pattern recreation using DTW wrapping path.

This concept is also visualized in Figure 1, where, in the left graph, the TF of the healthy structure (blue line) is affected by the damage (red dashed line) only at the first peak, with a difference *c*, while the rest remains unaffected. The error is also noticeable between the TF of the healthy structure and the TF of the FE model (orange line). On the right graph are the wrapped TFs of the FE model (dashed orange line) and the healthy structure (dashed blue line). However, using the wrapping path of the wrapped healthy TF, the reconstructed damaged TF (dotted red line) now creates the new pattern with the same difference as the real structure. The new pattern lowers the modeling error and allows the usage of the FE model within the context of the proposed methodology.

This procedure is made under the assumption that even if an error exists between the FE model and the healthy structure, similar damage on both the structure and the model will affect them in the same way. This will eventually create similar differences between the states of each one, meaning it will affect the same frequency range with a similar pattern.

The limitation of this assumption must be noted. In order to efficiently use the DTW, the TF curves of the healthy experimental structure and the FE model must follow a similar pattern even if their features are shifted on the frequency domain. If a feature, e.g., a peak in one curve, does not exist on the other curve, two scenarios are possible. Either it will be not wrapped efficiently and could result in an exclusion of this feature on the recreated curve, or it could also be wrapped on the next similar feature on the curve. In both scenarios, an error arises on the specific frequency range that creates a blind spot where, if the damage affects this specific frequency range, the proposed procedure might not able to find it.

**Figure 2:**
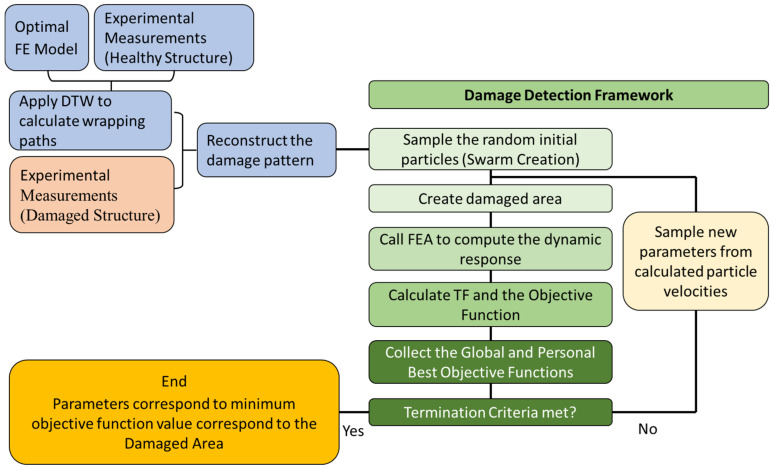
Flow chart of the proposed Damage Detection Framework.

In complex structures, not all the TF curves of the FE model might be able to be efficiently wrapped with the TF of the healthy structure. Thus, a selection must be made based on the quality of the wrapping between the curves and the quality of the recreated TF curve in order to include all the features. The procedure of selection is manual and should be carried out with caution. The flow chart of the entire proposed Damage Detection Framework is presented in Figure 2.

## 4. Experimental Setup and Validation

The present section describes the experimental setup, damage scenarios, and the application of the proposed methodology.

### 4.1. Experimental Setup

A CFRP truss was selected as an examined structure. It consists of CFRP filament-wound tubes where aluminum connectors are glued at each end. These connectors are bolted on intermediate aluminum parts. The complete structure is permanently clamped on a steel base which is attached to a concrete wall. The CFRP tubes consist of seven (7) plies, and the details are presented in Table 1.

**Figure 3:**
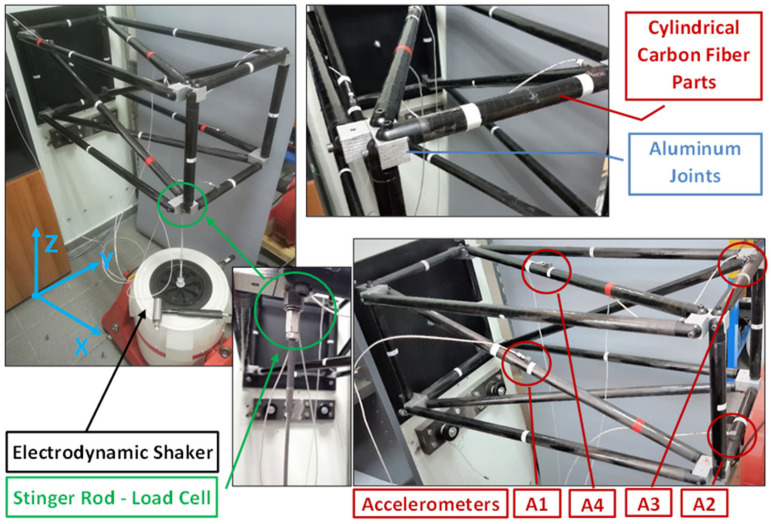
Experimental setup of the CFRP truss.

The measurement equipment consists of four triaxial accelerometers which are placed on different tubes of the truss. Below the truss, an electrodynamic shaker is connected in order to be used as an excitation source. The shaker is connected with a stinger rod and a bolted load cell on the part of the truss. For all experimental measurements, a sampling rate of 2048 Hz has been used and random Gaussian excitation was imposed on the shaker connected with the CFRP truss, as shown in Figure 3.

### 4.2. Finite Element Model

**Figure 4:**
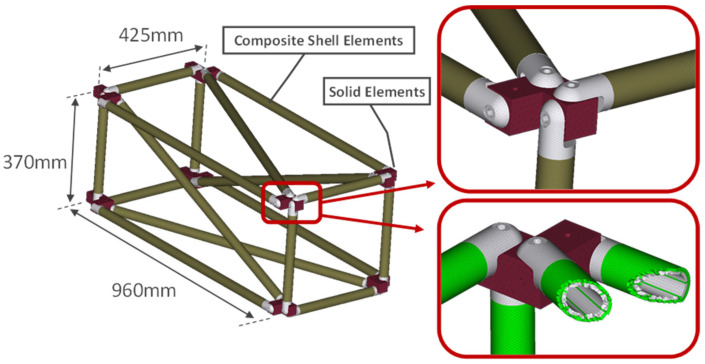
Finite Element model of the truss.

The FE model of the CFRP truss is shown in Figure 4. In total, the model is made of 1,480,912 elements (341,933 nodes) resulting in 2,051,598 degrees of freedom. The modeling of the aluminum joints, connections, and glue is performed with solid elements, while for the CFRP shell elements have been used. The pin-joint connections are modeled with rigid body elements between the aluminum joints and connections. The base of the four (4) aluminum joints in the back of the truss (with reference to Figure 4) are fully fixed, as the physical truss structure is permanently clamped on a steel base which is attached to the concrete wall. The random excitation is applied on the front low corner, at the same position the physical structure is connected with the electrodynamic shaker.

**Figure 5:**
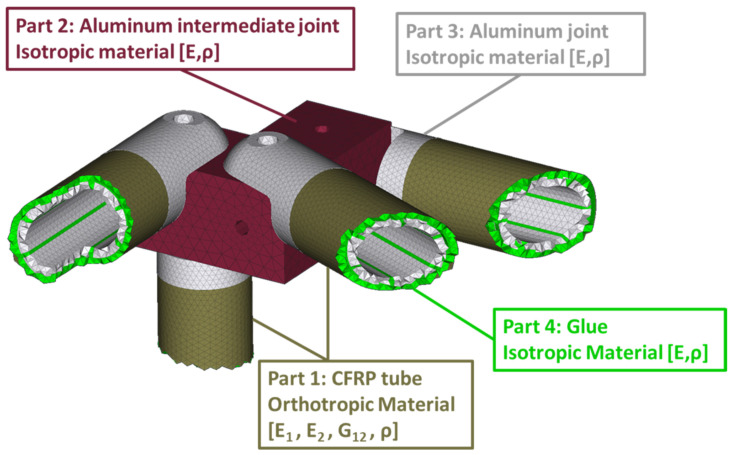
Parameterized FE model.

A detailed parametrization of the FE model is also depicted in Figure 5. Experimental measurements of healthy structures were used to obtain the Optimal FE model of the structure. A total of ten (10) material variables were included in the model updating process, using the CMA-ES algorithm with a comparison metric of Transmittance Functions.

The material parameters used for the Nominal FE model included the CFRP’s orthotropic material with $E_{1}=140.3\;GPa$, $E_{2}=8.6\;GPa$ for the modulus of elasticity in 1 (fiber) and 2 (matrix) direction, respectively, $\nu_{12}=0.27$ for the Poisson’s ratio, $G_{12}=4.61\;GPa$ for the in-plane transverse shear modulus, and $\rho =1525\;Kg/m^{3}$ for the density. The material parameters of the aluminum parts were set as E=68 GPa for the modulus of elasticity, ν=0.36 for the Poisson’s ratio, and $\rho =2698\;Kg/m^{3}$ for the density. The final updated parameters are presented in Table 2.

**Figure 6:**
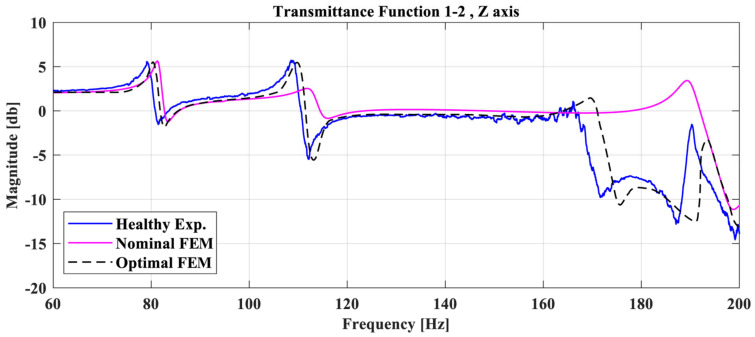
Comparison of TF between accelerometers 1,2 (Z-axis) between the Healthy experimental structure, the Nominal FE model, and the Optimal FE model.

Indicative Transmittance Functions (TFs) of the Nominal (purple line), Optimal FE model (dashed black line), and the Healthy experimental structure (blue line) between the accelerometer 1 and 2 on the Z-axis are presented in Figure 6. It is obvious that, after the model update process, there is still a modeling error rate for the Optimal FE model with the respective real experimental structure. To overcome this obstacle, the Dynamic Time Wrapping (DTW) algorithm will be used to allow the use of the FE model for damage detection purposes in the following sections.

### 4.3. Damage Cases

**Figure 7:**
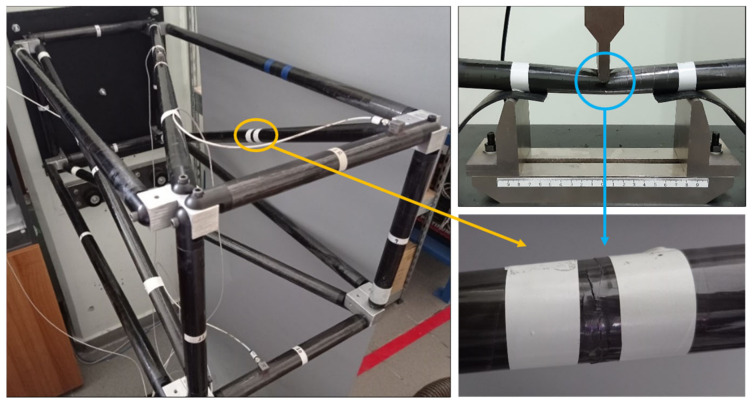
The damaged CFRP tube mounted on one of the four possible positions of the truss (**left**). Damage creation on the CFRP tube with the three-point bending system (**right**).

The experimental damage was created on a three-point bending scenario of a CFRP tube, Figure 7. The tube was placed in a compression machine while rubber material was placed under the two points that hold the tube, thus preventing unwanted damage in these regions. The resulting damage is a local reduction of stiffness, as multiple local cracks were created on the composite material.

**Figure 8:**
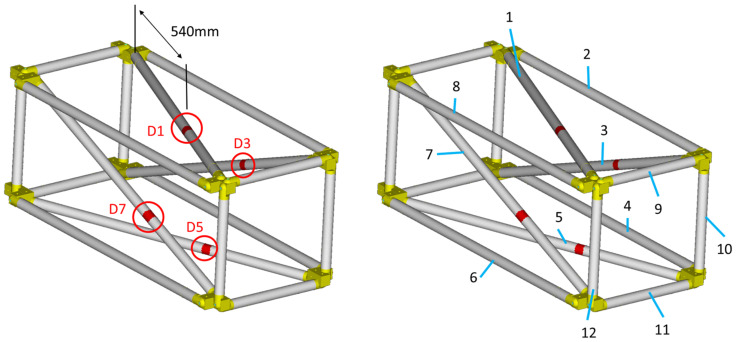
Damage cases (**left**). CFRP tube identification numbers (**right**).

In the current work, only single-part damage is examined, meaning only one instance of damage exists on the structure at each examined case. One diagonal tube of the truss was selected as the damaged part, which could be mounted in four different positions on the specific structure (tubes 1, 3, 5, 7), thus creating the four different damage cases: D1, D3, D5, and D7. The damage cases, along with the part numbers for the structure are presented in Figure 8. The four accelerometers that were mentioned in Section 4.1 are located on tubes 1, 7, 9, and 11.

**Figure 9:**
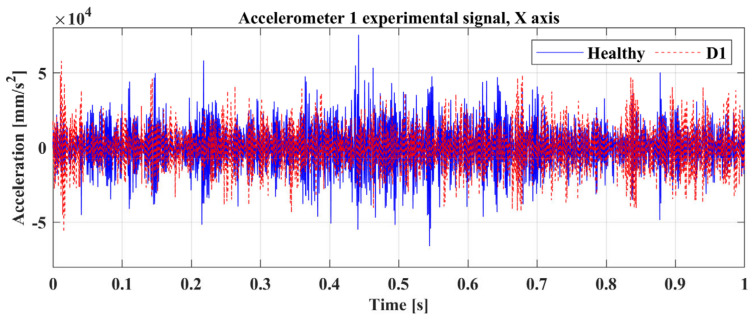
Experimental time response signal of Accelerometer 1 [A1] on X-axis.

Regarding the excitation on the structure, all experimental measurements were executed with a sampling rate of 2048 Hz and a random excitation signal from the electrodynamic shaker. Indicative experimental acceleration measurements are presented in Figure 9 from accelerometer 1 between the healthy state and damage case D1.

**Figure 10:**
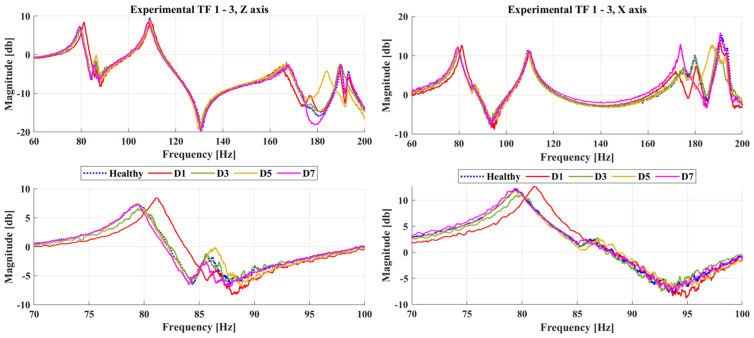
Experimental Transmittance Function between accelerometer 1 and 3 on the Z-axis (**left**) and X-axis (**right**).

Furthermore, Figure 10 shows some of the calculated TFs between accelerometers 1 and 3, including the healthy state and all damaged cases. In the given combinations of Transmittance Functions, the generated damage cases mainly affect the frequency range between 165 and 200 Hz, with some additional changes between 75 and 90 Hz.

### 4.4. Damage Detection

**Figure 11:**
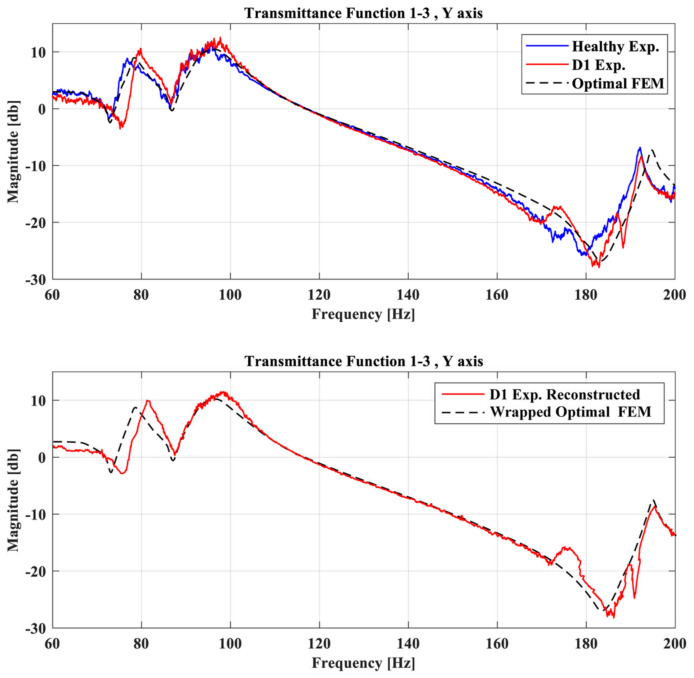
TFs of the Optimal FE model, healthy experimental and D1 case between accelerometer 1 and 3 at Y-axis (**up**), and TFs of the Optimal FE model and the reconstructed damage curve (**down**).

**Figure 12:**
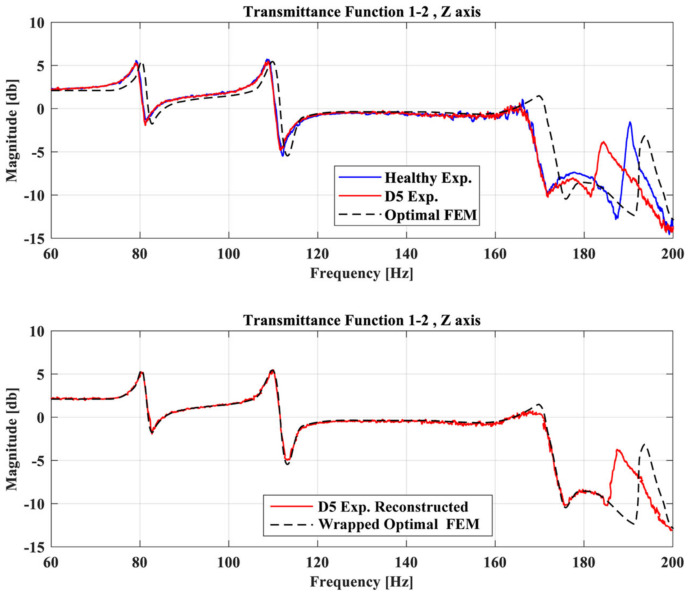
TFs of the Optimal FE model, healthy experimental and D5 case between accelerometer 1 and 2 at Z-axis (**up**), and TFs of the Optimal FE model and the reconstructed damage curve (**down**).

The first step of the procedure is to create the required wrapping path of the healthy TF and the FE Model for each TF separately. Figure 11 and Figure 12 show indicative TFs of two damage cases between the Optimal FE model (dashed black line), the structure at its healthy state (blue line), the damage state (red line), and also the reconstructed damaged TF curve. In both of these figures, it is obvious that a modeling error exists on the initial curves, and it would prevent the use of the damage detection framework, as it will probably indicate a false damaged area.

The pattern of the reconstructed damaged TF curve (red line) with the FE model (dashed black line) is similar to the pattern between the healthy (blue line) and damaged (red line) TF curves. Minor differences and errors on the new curves might appear, but if this procedure is completed carefully it will only add a small portion of anomalies compared with the initial modeling error. In the case that the initial curves show the same pattern but difference in magnitude, it is suggested that normalization will be applied to the data prior to the DTW.

All damage cases were executed using the range of [0.1, 1.5] for the material parameters $p_{E},p_{D}$. Furthermore, the composite tubes 1 to 12 (numbered in Figure 8) were used for the Part parameter as search space. The damaged area inserted into the FE model has a maximum length of 230 mm.

**Figure 13:**
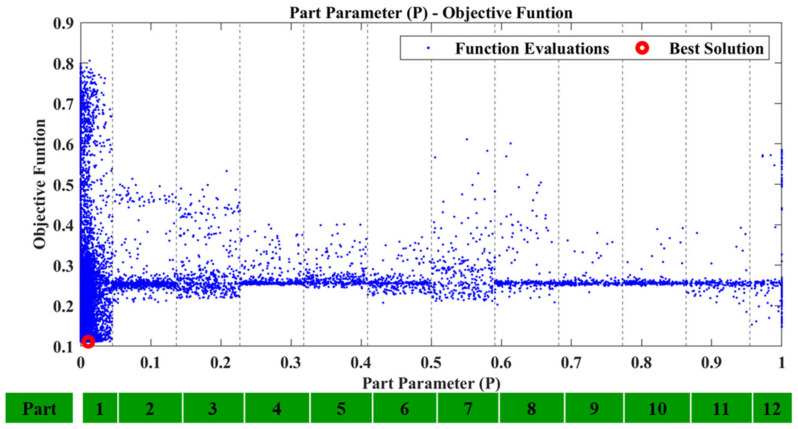
D1: Part parameter (P) versus the Objective Function values.

**Figure 14:**
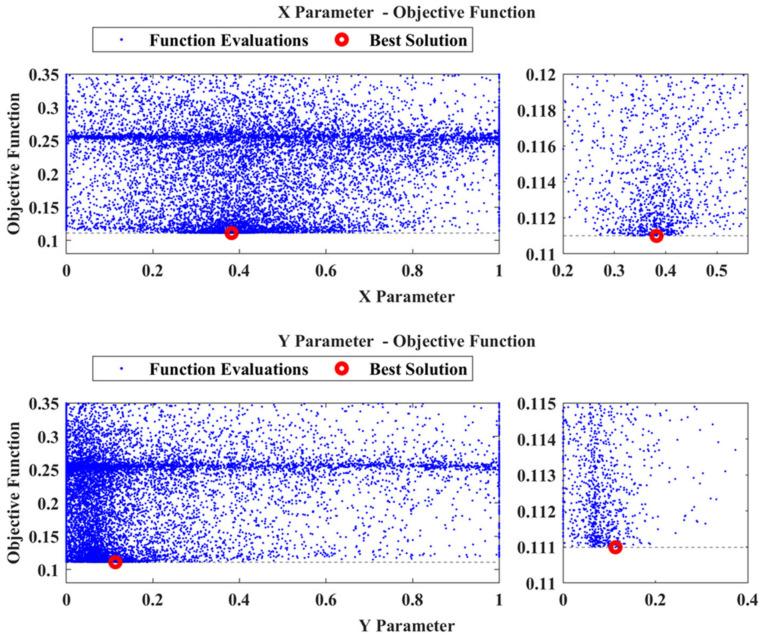
D1: X and Y parameters versus the Objective Function values.

Regarding the first case, D1, after executing the framework using the recreated damage curve the results were successful. The P parameter resulted inside the region which corresponds to Tube 1 and is presented in Figure 13. Figure 14 shows the locational parameters X and Y.

**Figure 15:**
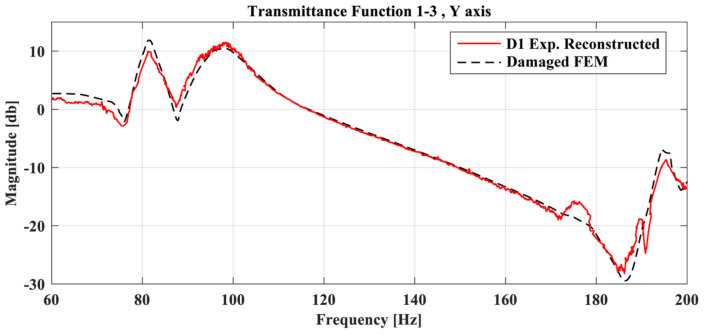
Transmittance Functions of the Damaged FE model and D1 case between accelerometer 1 and 3 at Y-axis.

As Tube 1 is expanding on the X, Y plane, the Z parameter does not have a pivotal role in the final damaged area. The final objective function had a value of 0.110989, with the material parameters $p_{E},p_{D}$ having a value of 0.767 and 1.016, respectively. As was mentioned, the proposed framework does not have the ability to quantify the damage as the material parameters depend on the size of the damaged area inserted into the FE model, but also on the modeling error that might still exist. As such, the values of $p_{E},p_{D}$ should not be interpreted as exact representations of the status of the tube. This is also the reason why the calculation of the exact material mechanical properties (e.g., modulus of elasticity and density) from the parameters $p_{E},p_{D}$ is not necessary. Moreover, Figure 15 shows the TF of the newly created damaged FE model (dashed black line) compared with the reconstructed TF curve of the D1 damage case (red line). Some differences are still present, which is expected, but it is obvious that the two curves have a higher linear correlation compared to before.

**Figure 16:**
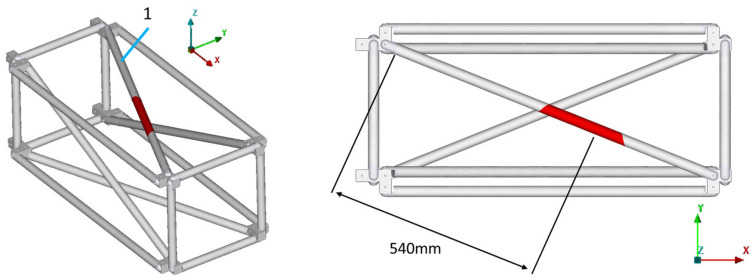
D1: Damaged FE model.

The final damaged FE model is presented in Figure 16, where the damaged area highlighted in red includes the damage of the physical structure.

**Figure 17:**
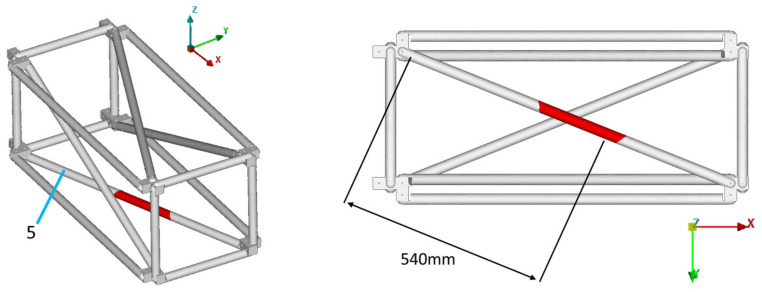
D5: Damaged FE model.

**Figure 18:**
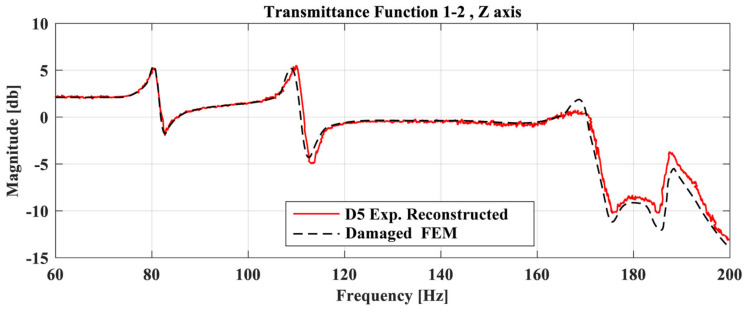
Transmittance Functions of the Damaged FE model and D5 case between accelerometer 1 and 3 at Y-axis.

**Figure 19:**
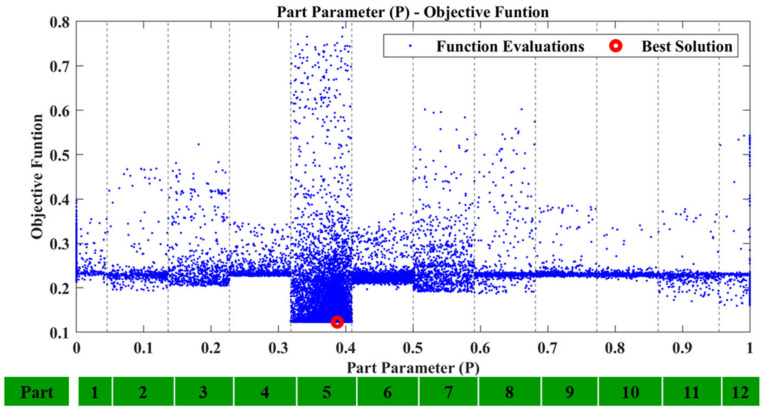
D5: Part parameter (P) versus the Objective Function values.

**Figure 20:**
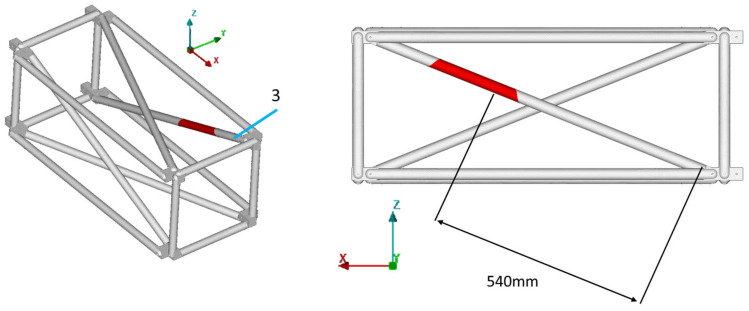
D3: Damaged FE model.

**Figure 21:**
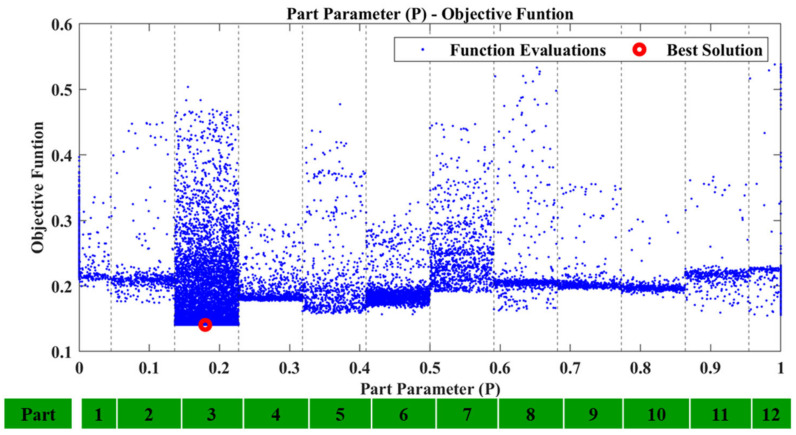
D3: Part parameter (P) versus the Objective Function values.

**Figure 22:**
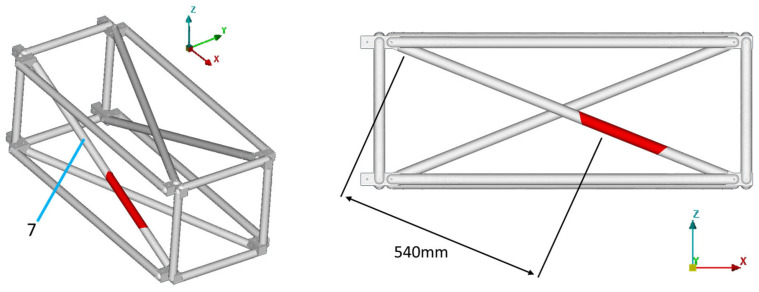
D7: Damaged FE model.

**Figure 23:**
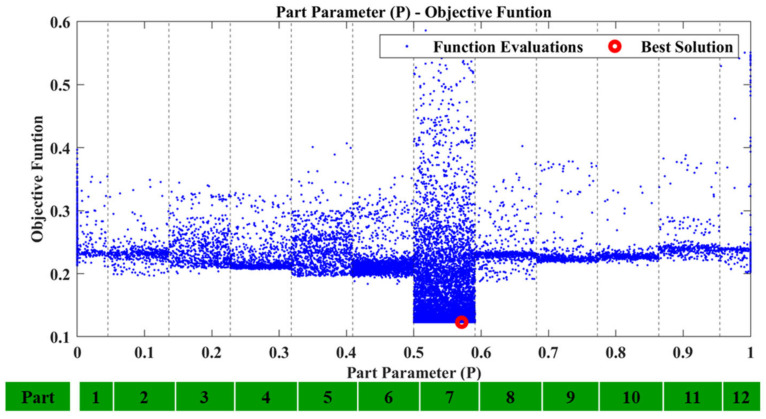
D7: Part parameter (P) versus the Objective Function values.

Equivalent results are obtained for the other damage cases. All the results for damage cases D3, D5, and D7 are summarized in Table 3, along with the corresponding Figure 17, Figure 18, Figure 19, Figure 20, Figure 21, Figure 22 and Figure 23 that showcase the damaged FE model and the part parameter for each case. The different combinations of the locational parameters were expected even with those large differences. The selected damaged tube is placed at an angle (diagonal tube) with the respected axis, and multiple combinations exist that result in the same damaged area. The examined cases were successful, and the physical damage is always included inside the model’s damaged area, highlighting the effectiveness of the proposed Damage Detection Framework.

Regarding the damaged case D5, Figure 18 shows the TFs of the damaged FE model and the experimental reconstructed curve of the D5 case. It is clear that the damage FE model has a high linear correlation with the reconstructed curve after the optimization procedure.

The results of the presented procedures show that, in all four damage cases, the framework was able to detect the structural damage. Additionally, the modeling error rate between the complex healthy structure and the Optimal FE model would not allow the framework to be used without the use of DTW. In conclusion, the framework can be applied without further processing of the TF curves if the modeling error is small, as already demonstrated in a simpler experimental setup, while in the case of complex structures where modeling error cannot be avoided, the use of DTW and reconstructed curves can provide a solution and allow the presented framework to be applied and the damage identified.

## 5. Conclusions

A methodology using a model-based damage detection method is presented using an FE model update procedure. The main goal is to advance the already existing method to be applicable to structures where the modeling error cannot be minimized. The development of an accurate FE model for simple structures may be an easy task, while in complex structures the modeling error may be unavoidable. In such cases, with a high rate of modeling error even after the optimization of the FE model, the previous version of the framework would not be accurate enough. To overcome this obstacle, a new procedure using the Dynamic Time Wrapping (DTW) algorithm is implemented in order to minimize the effect of the modeling error. DTW enables the damaged TF curve of the experimental structure to be reconstructed around the FE model curve. Therefore, the new damaged curve and the FE model have the same differences as the actual experimental damaged curve with the healthy experimental TF curve of the structure. In the case that the modeling error of the FE model is small, the framework can also be used without the application of DTW, as presented previously, although using DTW even with a small modeling error would increase the accuracy. In addition, limitations must also be addressed. Without the use of the DTW, the framework relies only on the accuracy of the optimal FE model, which may also be acceptable depending on the structure considered. If the DTW is used, then the modeling error is minimized, but even then the FE model curves must have similar characteristics (e.g., peaks and valleys) through the frequency domain. In addition, the framework can effectively locate the damaged area, but it cannot quantify the damage, as this work is also affected by other parameters, such as the size of the input damage in the FE model. Future research based on the proposed method could focus on other methods that could recreate the damaged pattern, but also the application to systems exhibiting non-linear behavior.

The effectiveness of the proposed framework is demonstrated on an experimental composite CFRP truss structure. As was shown, the peaks of the TF curves between the FE model and the healthy structure had a high percentage of error. While the error between individual peaks of the curves would reach a maximum value up to 4%, the accumulated error from all the curves and features of the structure would lead to a false indication of the damage. The recreation of the pattern allowed the wrapping of the curves in a non-linear manner and thus allowed all the important features of the curves to be kept while minimizing the accumulated error. Four cases of damage developed at different locations of the structure in the CFRP material. The framework, with the application of DTW, was able to successfully locate all four damage locations.

## Figures and Tables

**Table 1:** CFRP Composite Tube Properties.

Layer Orientation	(+/−8°) (+86°) (+/−8°) (+/−8°)	
Tube Internal Diameter	25 mm	
Layer Thickness	+/−8°	0.52 mm
	+86°	0.16 mm

**Table 2:** Updated FE model material properties.

Part			1			
Parameter			Bounds		Result	
Modulus of Elasticity, Direction 1, $E_{1}\;[\mathrm{GPa}]$			[90, 150]		110	
Modulus of Elasticity, Direction 2, $E_{2}\;[\mathrm{GPa}]$			[5.0, 10.0]		7.09	
In-plane Shear Modulus $G_{12}\;[\mathrm{GPa}]$			[4.0, 6.0]		5.34	
Density, ρ $\left[{\mathrm{kg}/m}^{3}\right]$			[1200, 1650]		1540	
**Part**	**2**		**3**		**4**	
**Parameter**	**Bounds**	**Result**	**Bounds**	**Result**	**Bounds**	**Result**
Young’s Modulus Ε [GPa]	[60, 80]	62.1	[60, 80]	75.8	[0.85, 1.15]	1.14
Density ρ $\left[{\mathrm{kg}/m}^{3}\right]$	[2450, 2950]	2482.5	[2450, 2950]	2650	[490, 1474]	983

**Table 3:** Parameter results for each damage case.

**Case**	**Parameters**						** Figures **	
	X	Y	Z	Part	$p_{E}$	$p_{D}$	**Damaged** **FE Model**	**Part** **Parameter**
D1	0.38	0.11	-	1	0.767	1.016	Figure 16	Figure 13
D5	0.54	0.50	-	5	0.730	1.023	Figure 17	Figure 19
D3	0.73	-	0.60	3	0.702	1.100	Figure 20	Figure 21
D7	0.63	-	0.81	7	0.780	0.970	Figure 22	Figure 23

## Data Availability

Data available on request due to restrictions. The data presented in this study are available on request from the corresponding author. The data are not publicly available due to privacy restrictions of ongoing research.
